# Preliminary Results on a Cost-Effectiveness Analysis of the ACHIEVE Hearing Intervention

**DOI:** 10.1093/geroni/igaf122.1624

**Published:** 2025-12-31

**Authors:** Emmanuel Garcia Morales, Victoria Sanchez, Haley Calloway, Laura Sherry, Michelle Arnold, James Pike, Nicholas Reed

**Affiliations:** New York University Langone Health, New York, New York, United States; University of South Florida, Tampa, Florida, United States; University of South Florida, Tampa, Florida, United States; Johns Hopkins University School of Medicine, Baltimore, Maryland, United States; University of South Florida, Tampa, Florida, United States; New York University, New York, New York, United States; New York University Grossman School of Medicine, New York, New York, United States

## Abstract

This study evaluates the cost-effectiveness of a best-practices hearing intervention in mitigating cognitive decline, measured by Mini-Mental State Examination (MMSE) scores, over three years in the ACHIEVE trial (NCT03243422). ACHIEVE, a randomized controlled trial, compared a hearing intervention to a successful aging health education control group among older adults with hearing loss. We enrolled 977 adults (70-84 years) with untreated mild-to-moderate hearing loss from four U.S. sites. Participants were randomized to the intervention (N = 490) or control group (N = 487). The intervention included audiologist-fitted hearing aids, assistive devices, counseling, and ongoing support, while the control group received health education on aging. Costs were assessed from the provider’s perspective, covering professional services and technology. Three years post-randomization, MMSE scores declined less in the intervention group by 0.28 points (95% CI: 0.01-0.55). The intervention cost an average of $3,900.53 per participant, including $692.22 for professional services and $3,208.31 for technology. The incremental cost-effectiveness ratio (ICER) was estimated at $13,917.76 per MMSE point preserved. The ACHIEVE intervention, a non-pharmacologic, low-risk approach, showed a modest cognitive benefit at a cost of ∼$14K per MMSE point over three years. Comparatively, anti-amyloid drug treatments have reported an ICER of $60,516.82/MMSE over 76 weeks. These findings support the potential role of hearing interventions in cognitive health strategies and inform evidence-based decision-making.

